# Supplementary material for: Convergent Evolution During Local Adaptation to Patchy Landscapes
Source: PLoS Genet. 2015 Nov 16;11(11):e1005630. doi: 10.1371/journal.pgen.1005630 (PMC4646681; doi:10.1371/journal.pgen.1005630)
Supplement: S1 Text — (PDF) [file pgen.1005630.s001.pdf]

Supporting Information

The exact solution to the equilibrium frequency

Here we describe how to solve (24) exactly in one dimension, i.e., the Fisher-KPP equation with piecewise constant selection. Let $p(t,x)$ be the proportion of the allele under spatially varying selection $s(x)$ in one dimension, so that

$$\partial_t p(t,x) = \frac{\mu}{2}\sigma^2 \partial_x^2 p(t,x) + \mu s(x) p(t,x)(1 - p(t,x)).$$

The stable distribution $\phi(x) = \lim_{t\to\infty} p(t,x)$ then solves

$$\partial_x^2 \phi(x) = -2s(x)\phi(x)(1-\phi(x))/\sigma^2, \tag{60}$$

with appropriate boundary conditions. First rescale space by $\sigma/\sqrt{2}$ so the $\sigma^2/2$ term disappears. If we now assume that $s(x)$ is piecewise constant, $s(x) = s_i$ for $x \in [x_i, x_{i+1})$, with $x_0 = -\infty$ and $x_{n+2} = \infty$, then the equation is integrable: if we multiply through by $2\partial_x \phi(x)$ and integrate, then we get that

$$\begin{aligned}
(\partial_x \phi(x))^2 &= -\int^x 2s(x)\phi(x)(1-\phi(x))\partial_x\phi(x)dx && (61)\\
&= -s_i \phi(x)^2\left(1 - \frac{2}{3}\phi(x)\right) - K_i \quad \text{for } x \in [x_i, x_{i+1}), && (62)
\end{aligned}$$

if we define

$$K_i = -s_i\phi(x_i)^2\left(1 - \frac{2}{3}\phi(x_i)\right) - (\partial_x\phi(x_i))^2. \tag{63}$$

For ease of reference, we define

$$V_i(\phi) = s_i\phi^2\left(1 - \frac{2}{3}\phi\right) + K_i.$$

Note that $V_i'(0) = V_i'(1) = 0$, that $V_i(0) = K_i$ and $V_i(1) = K_i + s_i/3$. We will always have that $V(\phi) \le 0$. (We have then that $\partial_x^2\phi = -\partial_\phi V(\phi)$, the equation of motion of a particle in potential V. Also note that this implies "conservation of energy", i.e., $(\partial_x\phi(x))^2 + V(\phi(x))$ is constant.) Rearranging, we get that $dx = d\phi/\sqrt{-V(\phi)}$, so where $\phi(x)$ is monotone, the inverse is

$$x(\phi) = x(\phi^*) \pm \int_{\phi^*}^{\phi} \frac{d\psi}{\sqrt{-V(\psi)}}.$$

For each i then define the elliptic function

$$F_i(\phi) = \int_{\phi_i^*}^{\phi} \frac{d\psi}{\sqrt{-V_i(\psi)}}, \tag{64}$$

where take the positive branch of the square root, and ϕ_i^* will be chosen later. Then we have that $x(\phi) - x(\phi_0) = \pm(F(\phi) - F(\phi_0))$, or for an appropriate x_0,

$$\phi(x) = F^{-1}\left(F(\phi(x_0)) \pm (x - x_0)\right).$$

We clearly want $\lim_{x\to\infty}\partial_x\phi(\pm x) = 0$, and $\lim_{x\to\infty}\phi(\pm x)$ to be zero or one depending on the sign of s_0 and s_{n+1}. Since $(\partial_x\phi(x))^2 = -V(x)$, this implies that if $s_0 < 0$, then $K_0 = 0$, while if $s_0 > 0$ then $K_0 = s_0/6$; and likewise for K_{n+1}.

We also require that $\phi(x)$ and $\phi'(x)$ are continuous. Continuity of $\phi'(x)$ is equivalent to $V_i(x_{i+1}) = V_{i+1}(x_{i+1})$, which we can rearrange to find an equation for K_{i+1} in terms of K_i and $\phi(x_{i+1})$:

$$K_{i+1} - K_i = (s_i - s_{i+1})\phi(x_{i+1})^2(1 - 2\phi(x_{i+1})/3).$$

What about the frequency at the points the selection changes, $\phi(x_i)$? Well, if $\phi(x)$ is monotone on $[x_i, x_{i+1})$ then we can without loss of generality take $\phi_i^* = 0$ or 1 depending on the sign of s_i. Otherwise, let ϕ_i^* be the (unique) root of V_i in $[x_i, x_{i+1})$, so $V_i(\phi_i^*) = 0$. In this case, ϕ_i^* is the maximum or minimum of ϕ in the interval: if $s > 0$, then $\phi_i = \max\{\phi(x) : x \in [x_i, x_{i+1})\}$. Recall we defined F_i using ϕ_i^*; now using the fact that ϕ is monotone with the opposite sign on either side of ϕ_i^*, $x_{i+1}-x_i = F_i(\phi(x_{i+1}))-F_i(\phi_i^*)+F_i(\phi(x_i))-F_i(\phi_i^*)$, and that $F(\phi_i^*) = 0$, we know that the length of the ith stretch is

$$x_{i+1} - x_i = \pm\left(F_i(\phi(x_i)) + F_i(\phi(x_{i+1}))\right).$$

Note that all the $\pm$'s are easily relatable to the signs of s_i. If we knew $\phi(x_1)$ and $\phi'(x_1)$, then we'd be able to solve the equations for $\phi(x_i)$ and K_i recursively upwards. In some cases, such as [9], we can infer $\phi(0)$ and $\partial_x\phi(0)$ by spatial symmetry. In other cases, we are only given $\phi(-\infty)$ and $\phi(\infty)$, and have to work inwards from the ends.

Doing the integrals

An important ingredient in the above method is the integral (64), to which a method for solving the Korteweg-de Vries equation can be applied [37]. Recall that $V_i(\phi) = s_i\phi^2(1 - 2\phi/3) + K_i$, with $K_i = V_i(0)$ chosen to match V at the boundaries; since we're just working within an interval with s constant, we can rescale space by $\sqrt{2s_i}/\sigma$, so that s and σ drop from the equation. We then want to integrate

$$\int \frac{d\phi}{\sqrt{-V(\phi)}} = \int \frac{d\phi}{\sqrt{\phi^2(1-2\phi/3)+K}},$$

over a domain where V is always negative. Let $\phi^2(1 - 2\phi/3) + K = (\alpha - \phi)(\phi - \beta)(\phi - \gamma)$, and change variables first to $y^2 = (\alpha - \phi)$, and then to $x = y/\sqrt{\alpha - \beta}$, so that

$$\begin{aligned}\frac{d\phi}{\sqrt{\phi^2(1-2\phi/3)+K}} &= \frac{-2dy}{\sqrt{(\alpha-\beta-y^2)(\alpha-\gamma-y^2)}} \\ &= \frac{-2dx}{\sqrt{\alpha-\gamma}\sqrt{(1-x^2)(1-S^2x^2)}},\end{aligned}$$

with $S^2 = (\alpha - \beta)/(\alpha - \gamma)$. Now Jacobi's incomplete elliptic integral of the first kind is defined by

$$F(x;k) = \int_0^x \frac{dt}{\sqrt{(1-t^2)(1-k^2t^2)}},$$

and the Jacobian elliptic function $\mathrm{sn}(x;k)$ is the inverse: $F(\mathrm{sn}(x;k);k) = x$. As $k \to 0$, $\mathrm{sn}(x;k) \to \sin(x)$, while as $k \to 1$, $\mathrm{sn}(x;k) \to \sinh(x)$.

More information about the simulations

In tables S1 and S2 we provide additional details regarding the simulations we used to validate the theory in figures 4 and 5.
