# Supplementary material for: Convergent Evolution During Local Adaptation to Patchy Landscapes
Source: PLoS Genet. 2015 Nov 16;11(11):e1005630. doi: 10.1371/journal.pgen.1005630 (PMC4646681; doi:10.1371/journal.pgen.1005630)
Supplement: S1 Table — All simulations also used a linear grid of 501 demes with a patch of 99 demes in the center, the migration model described in Simulation methods, μ = 10−5, and s p = .0023 (calculated as the growth rate as described in the text). T adapt is the mean time until 100 B alleles were present in the patch, and p adapted is the proportion of the simulations that adapted by the 25,000 generations. The simulation began with no B alleles. (PDF) [file pgen.1005630.s003.pdf]

μ	ρ	s_p	s_m	T_{adapt}	p_{adapted}	n	μ	ρ	s_p	s_m	T_{adapt}	p_{adapted}	n
1e-05	25	0.01	-0.10	19630	0.06	100	1e-05	600	0.01	-0.10	1082	1.00	40
1e-05	25	0.01	-0.01	15533	0.55	100	1e-05	600	0.01	-0.01	924	1.00	40
1e-05	25	0.01	-0.00	14353	0.72	100	1e-05	600	0.01	-0.01	828	1.00	40
1e-05	25	0.01	-0.00	13282	0.74	100	1e-05	600	0.01	-0.00	846	1.00	40
1e-05	50	0.01	-0.10	11587	0.33	40	1e-05	600	0.01	-0.00	966	1.00	40
1e-05	50	0.01	-0.10	11586	0.38	40	1e-05	800	0.01	-0.10	802	1.00	40
1e-05	50	0.01	-0.01	9419	0.88	40	1e-05	800	0.01	-0.10	1182	1.00	40
1e-05	50	0.01	-0.01	8848	0.82	40	1e-05	800	0.01	-0.01	846	1.00	40
1e-05	50	0.01	-0.00	6529	0.95	40	1e-05	800	0.01	-0.01	607	1.00	40
1e-05	50	0.01	-0.00	9986	0.90	40	1e-05	800	0.01	-0.00	646	1.00	40
1e-05	100	0.01	-0.10	7425	0.91	316	1e-05	800	0.01	-0.00	507	1.00	40
1e-06	100	0.01	-0.10	21010	0.23	217	1e-05	1000	0.01	-0.10	615	1.00	256
1e-05	100	0.01	-0.03	6036	0.98	195	2.4e-06	1000	0.01	-0.10	5309	0.96	257
1e-06	100	0.01	-0.03	19859	0.31	194	1e-05	1000	0.01	-0.03	523	1.00	194
1e-05	100	0.01	-0.01	5408	0.98	315	1e-06	1000	0.01	-0.03	6425	0.95	195
1e-06	100	0.01	-0.01	20606	0.28	216	1e-05	1000	0.01	-0.01	449	1.00	254
1e-05	100	0.01	-0.00	4745	0.99	195	2.4e-06	1000	0.01	-0.01	4806	0.96	256
1e-06	100	0.01	-0.00	19564	0.33	195	1e-05	1000	0.01	-0.00	460	1.00	193
1e-05	100	0.01	-0.00	4894	0.99	315	1e-06	1000	0.01	-0.00	5203	0.98	194
1e-06	100	0.01	-0.00	19417	0.33	216	1e-05	1000	0.01	-0.00	444	1.00	255
1e-05	100	0.01	-0.00	4940	0.99	316	2.4e-06	1000	0.01	-0.00	4037	1.00	257
1e-06	100	0.01	-0.00	18025	0.40	217	1e-05	1000	0.01	-0.00	404	1.00	216
1e-05	200	0.01	-0.10	4296	0.97	40	1e-06	1000	0.01	-0.00	4567	1.00	216
1e-05	200	0.01	-0.10	3736	1.00	40	1e-05	1200	0.01	-0.10	501	1.00	40
1e-05	200	0.01	-0.01	2943	1.00	40	1e-05	1200	0.01	-0.10	460	1.00	40
1e-05	200	0.01	-0.01	2402	1.00	40	1e-05	1200	0.01	-0.01	336	1.00	40
1e-05	200	0.01	-0.00	1899	1.00	40	1e-05	1200	0.01	-0.01	431	1.00	40
1e-05	200	0.01	-0.00	1992	1.00	40	1e-05	1200	0.01	-0.00	390	1.00	40
1e-05	400	0.01	-0.10	1740	1.00	100	1e-05	1200	0.01	-0.00	372	1.00	40
1e-05	400	0.01	-0.01	1471	1.00	100	1e-05	1600	0.01	-0.10	299	1.00	100
1e-05	400	0.01	-0.00	1446	1.00	100	1e-05	1600	0.01	-0.01	462	0.99	100
1e-05	400	0.01	-0.00	1224	1.00	100	1e-05	1600	0.01	-0.00	241	1.00	100
1e-05	600	0.01	-0.10	1276	1.00	40	1e-05	1600	0.01	-0.00	274	1.00	100

Table S1. Parameter values used in estimates of mean time to adaptation by mutation of figure 4. All simulations also used a linear grid of 501 demes with a patch of 99 demes in the center, the migration model described in Simulation methods, $\mu = 10^{-5}$, and $s_p = .0023$ (calculated as the growth rate as described in the text). T_{adapt} is the mean time until 100 B alleles were present in the patch, and p_{adapted} is the proportion of the simulations that adapted by the 25,000 generations. The simulation began with no B alleles.
