# Supplementary material for: Convergent Evolution During Local Adaptation to Patchy Landscapes
Source: PLoS Genet. 2015 Nov 16;11(11):e1005630. doi: 10.1371/journal.pgen.1005630 (PMC4646681; doi:10.1371/journal.pgen.1005630)
Supplement: S2 Table — All simulations also used a linear grid of 501 demes with two patches of 99 demes each, separated by R demes in the center; the migration model described in Simulation methods, μ = 10−5, and s p = .0023 (calculated as the growth rate as described in the text). T adapt is the mean time until 100 B alleles were present in the patch, and p adapted is the proportion of the simulations that adapted by the 25,000 generations. At the start of the simulation, one patch was initialized with a frequency of 0.8 B alleles, which were absent elsewhere. (PDF) [file pgen.1005630.s004.pdf]

R	ρ	s_p	s_m	T_{adapt}	p_{adapted}	n	R	ρ	s_p	s_m	T_{adapt}	p_{adapted}	n
100	100	0.01	-1e-01	24975	0.00	300	60	1000	0.01	-3e-02	22224	0.21	200
10	100	0.01	-1e-01	24975	0.00	40	100	1000	0.01	-1e-02	18098	0.55	299
120	100	0.01	-1e-01	24975	0.00	295	10	1000	0.01	-1e-02	14	1.00	100
160	100	0.01	-1e-01	24975	0.00	40	120	1000	0.01	-1e-02	23242	0.14	297
20	100	0.01	-1e-01	24975	0.00	40	160	1000	0.01	-1e-02	24762	0.01	100
40	100	0.01	-1e-01	24975	0.00	40	20	1000	0.01	-1e-02	136	1.00	100
60	100	0.01	-1e-01	24975	0.00	300	40	1000	0.01	-1e-02	874	1.00	99
80	100	0.01	-1e-01	24975	0.00	40	60	1000	0.01	-1e-02	3485	1.00	300
100	100	0.01	-3e-02	24975	0.00	200	80	1000	0.01	-1e-02	9204	0.96	100
120	100	0.01	-3e-02	24975	0.00	199	100	1000	0.01	-3e-03	4168	1.00	200
60	100	0.01	-3e-02	24903	0.01	200	120	1000	0.01	-3e-03	6304	1.00	200
100	100	0.01	-1e-02	24354	0.04	300	60	1000	0.01	-3e-03	1273	1.00	199
10	100	0.01	-1e-02	1705	0.95	40	100	1000	0.01	-1e-03	2813	1.00	299
120	100	0.01	-1e-02	24802	0.01	299	10	1000	0.01	-1e-03	12	1.00	100
160	100	0.01	-1e-02	24975	0.00	40	120	1000	0.01	-1e-03	4002	1.00	300
20	100	0.01	-1e-02	3601	0.95	40	160	1000	0.01	-1e-03	7386	1.00	100
40	100	0.01	-1e-02	9463	0.88	40	20	1000	0.01	-1e-03	121	1.00	100
60	100	0.01	-1e-02	18674	0.45	299	40	1000	0.01	-1e-03	507	1.00	100
80	100	0.01	-1e-02	23899	0.12	40	60	1000	0.01	-1e-03	1115	1.00	300
100	100	0.01	-3e-03	14601	0.80	200	80	1000	0.01	-1e-03	1941	1.00	100
120	100	0.01	-3e-03	18645	0.53	199	100	1000	0.01	-1e-04	2528	1.00	300
60	100	0.01	-3e-03	5424	0.98	200	10	1000	0.01	-1e-04	11	1.00	99
100	100	0.01	-1e-03	8992	0.96	299	120	1000	0.01	-1e-04	3590	1.00	298
10	100	0.01	-1e-03	522	1.00	40	160	1000	0.01	-1e-04	5648	1.00	100
120	100	0.01	-1e-03	11375	0.93	300	20	1000	0.01	-1e-04	122	1.00	100
160	100	0.01	-1e-03	16930	0.68	40	40	1000	0.01	-1e-04	498	1.00	100
20	100	0.01	-1e-03	1518	0.97	40	60	1000	0.01	-1e-04	1051	1.00	298
40	100	0.01	-1e-03	2234	1.00	40	80	1000	0.01	-1e-04	1796	1.00	100
60	100	0.01	-1e-03	4277	0.98	300	10	4000	0.01	-1e-01	0	1.00	20
80	100	0.01	-1e-03	5328	1.00	40	160	4000	0.01	-1e-01	24975	0.00	20
100	100	0.01	-1e-04	6867	0.99	299	20	4000	0.01	-1e-01	4406	1.00	20
10	100	0.01	-1e-04	461	1.00	40	40	4000	0.01	-1e-01	24975	0.00	20
120	100	0.01	-1e-04	9347	0.99	300	80	4000	0.01	-1e-01	24975	0.00	20
160	100	0.01	-1e-04	12731	0.90	40	10	4000	0.01	-1e-02	3746	0.85	20
20	100	0.01	-1e-04	860	1.00	40	160	4000	0.01	-1e-02	24975	0.00	20
40	100	0.01	-1e-04	2031	1.00	40	20	4000	0.01	-1e-02	58	1.00	20
60	100	0.01	-1e-04	3529	0.99	300	40	4000	0.01	-1e-02	362	1.00	20
80	100	0.01	-1e-04	4839	1.00	40	80	4000	0.01	-1e-02	4025	1.00	20
100	1000	0.01	-1e-01	24975	0.00	300	10	4000	0.01	-1e-03	6244	0.75	20
10	1000	0.01	-1e-01	1004	0.99	100	160	4000	0.01	-1e-03	4399	1.00	20
120	1000	0.01	-1e-01	24975	0.00	298	20	4000	0.01	-1e-03	52	1.00	20
160	1000	0.01	-1e-01	24975	0.00	100	40	4000	0.01	-1e-03	281	1.00	20
20	1000	0.01	-1e-01	18403	0.42	100	80	4000	0.01	-1e-03	1209	1.00	20
40	1000	0.01	-1e-01	24965	0.00	100	10	4000	0.01	-1e-04	3746	0.85	20
60	1000	0.01	-1e-01	24975	0.00	300	160	4000	0.01	-1e-04	3798	1.00	20
80	1000	0.01	-1e-01	24975	0.00	100	20	4000	0.01	-1e-04	52	1.00	20
100	1000	0.01	-3e-02	24975	0.00	200	40	4000	0.01	-1e-04	279	1.00	20
120	1000	0.01	-3e-02	24975	0.00	200	80	4000	0.01	-1e-04	1039	1.00	20

Table S2. Parameter values used in estimates of mean time to adaptation by migration of figure 4. All simulations also used a linear grid of 501 demes with two patches of 99 demes each, separated by R demes in the center; the migration model described in Simulation methods, $\mu = 10^{-5}$, and $s_p = .0023$ (calculated as the growth rate as described in the text). T_{adapt} is the mean time until 100 B alleles were present in the patch, and p_{adapted} is the proportion of the simulations that adapted by the 25,000 generations. At the start of the simulation, one patch was initialized with a frequency of 0.8 B alleles, which were absent elsewhere.
