# Supplementary figures and images for: Convergent Evolution During Local Adaptation to Patchy Landscapes
Source: PLoS Genet. 2015 Nov 16;11(11):e1005630. doi: 10.1371/journal.pgen.1005630 (PMC4646681; doi:10.1371/journal.pgen.1005630)

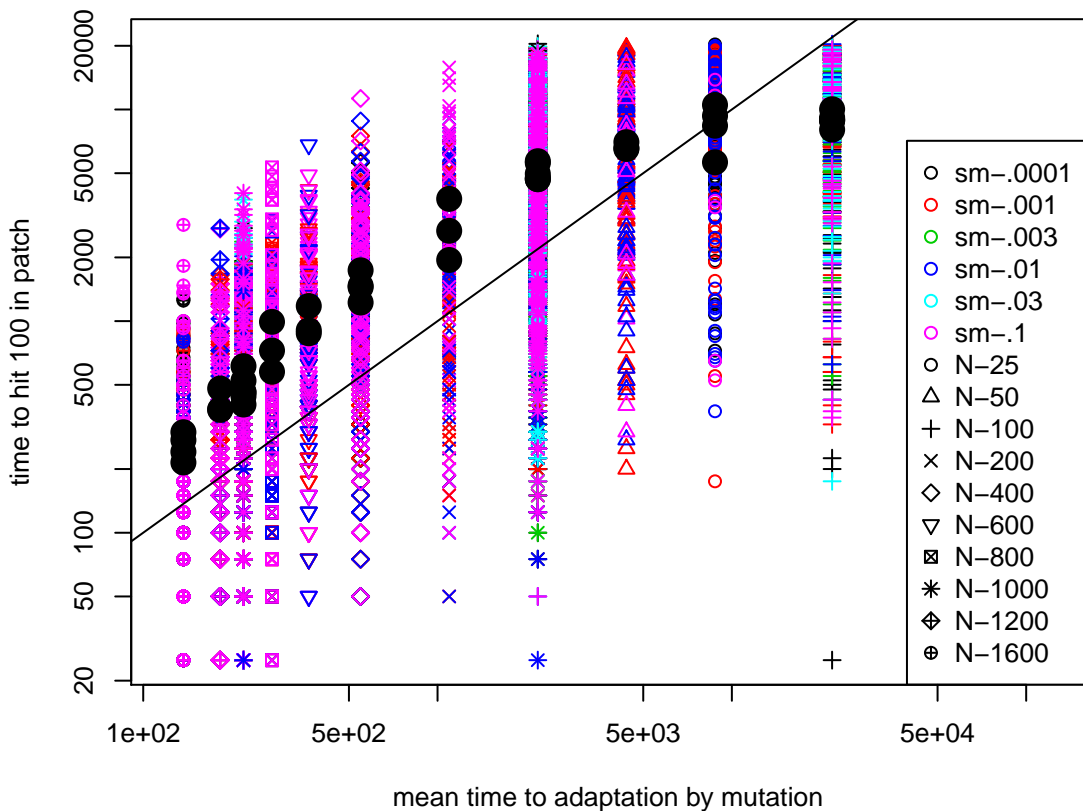

Supplement: S1 Fig — The same data shown in the left panel of Fig 4, but all times shown (not just the interquartile ranges), and including those parameter values at which most of the simulations did not adapt by 25,000 generations. (PDF) [file pgen.1005630.s005.pdf]

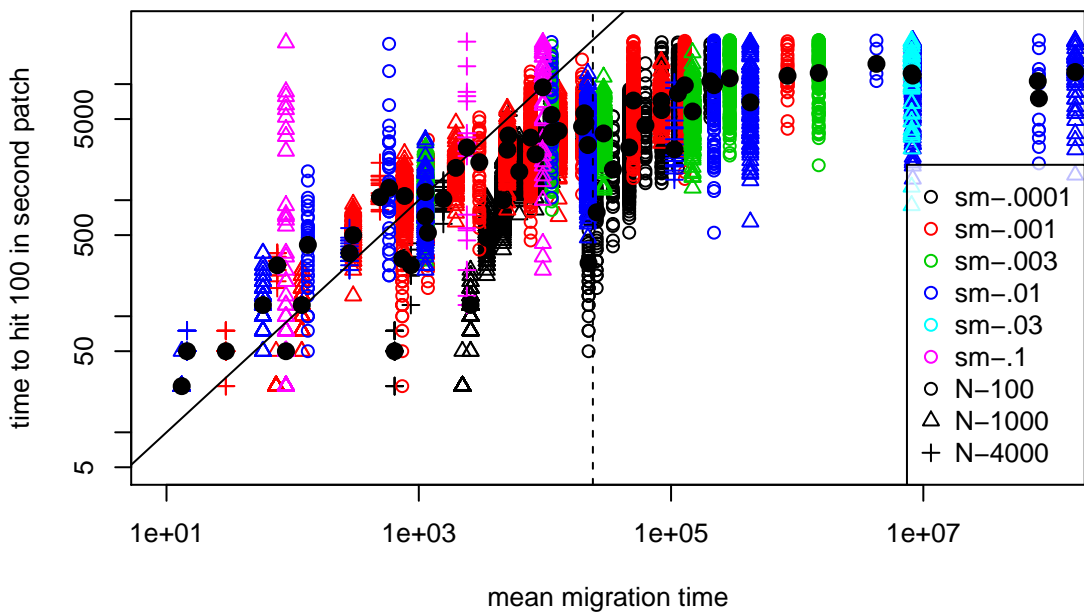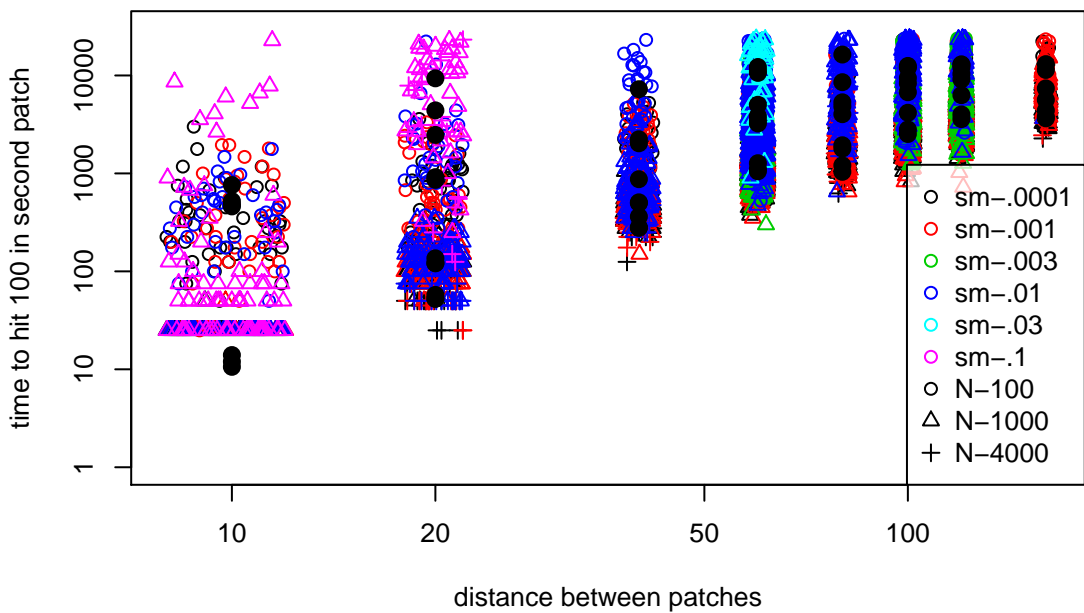

Supplement: S2 Fig — The same data shown in the right panel of Fig 4, but all times shown (not just the interquartile ranges), and including those parameter values at which most of the simulations did not adapt by 25,000 generations. The upper panel has the predicted time to adaptation on the horizontal axis as in Fig 4, and the lower panel has, for comparison, the raw distance between patches (which predicts time to adaptation, but not as well). (PDF) [file pgen.1005630.s006.pdf]

$\rho = 1200, \mu = 1e-05$

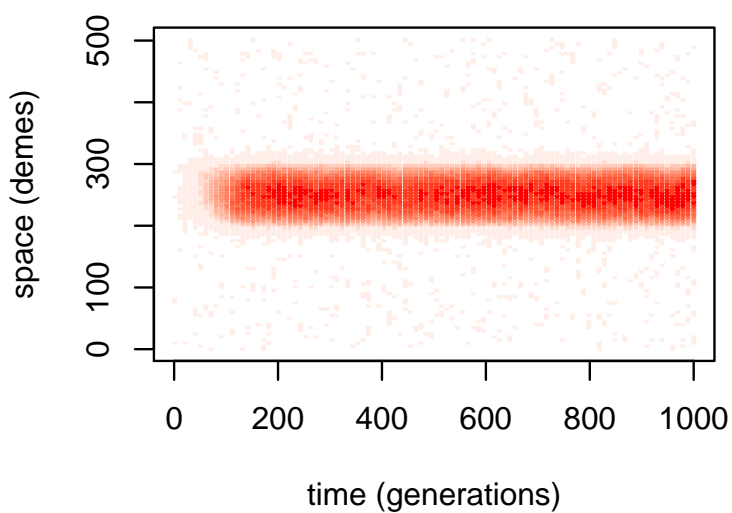

$s_m = 0.1, s_p = 0.00231$

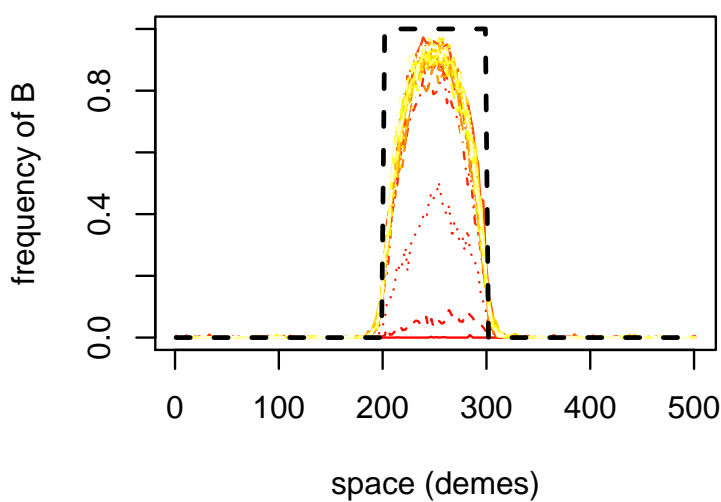

$\rho = 600, \mu = 1e-05$

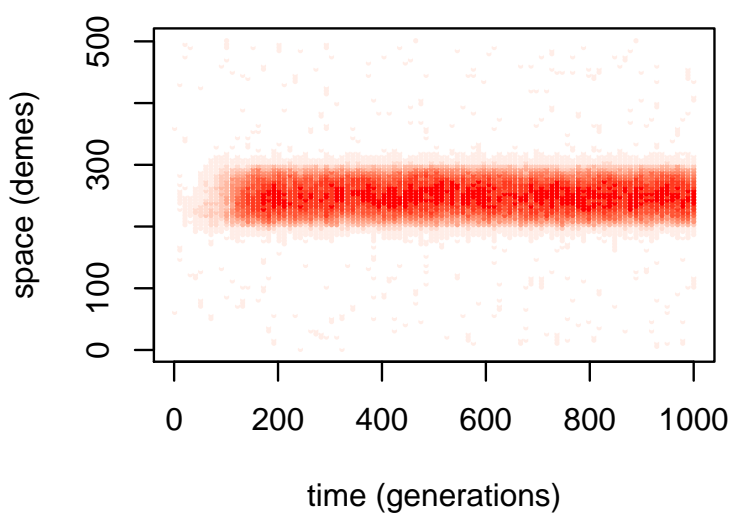

$s_m = 0.1, s_p = 0.00231$

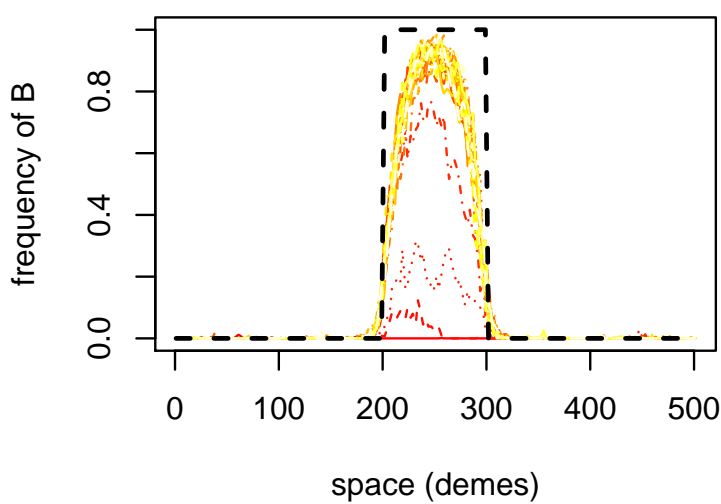

$\rho = 50, \mu = 1e-05$

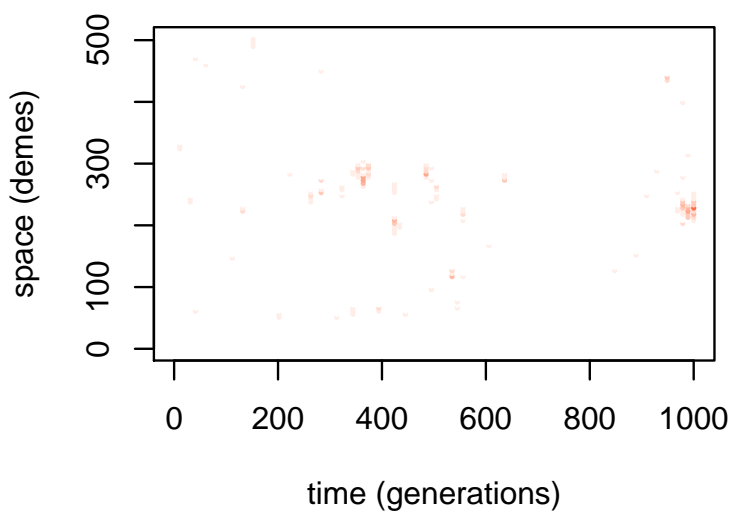

$s_m = 0.1, s_p = 0.00231$

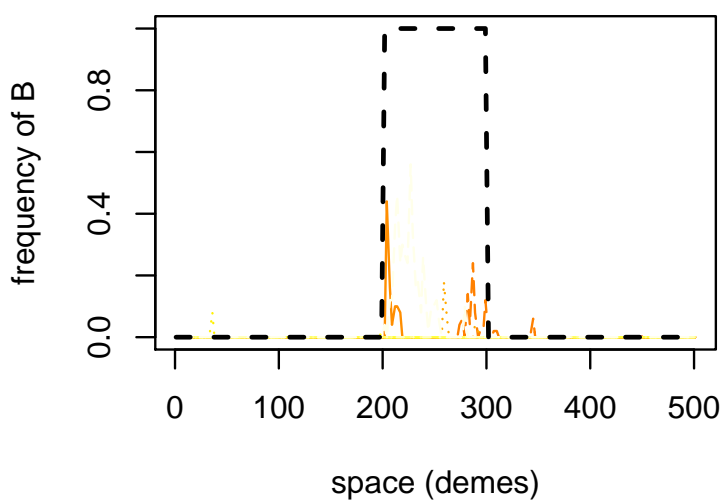

Supplement: S3 Fig — On the left of each is a space-time heatmap of the local frequency of B alleles; and on the right are twenty-five curves showing the frequencies of B at evenly spaced time points (i.e., each line represents a vertical slice through the plot on the left); dotted black lines indicate the patches where B is advantageous. (PDF) [file pgen.1005630.s007.pdf]

$\rho = 1200, \mu = 1e-05$

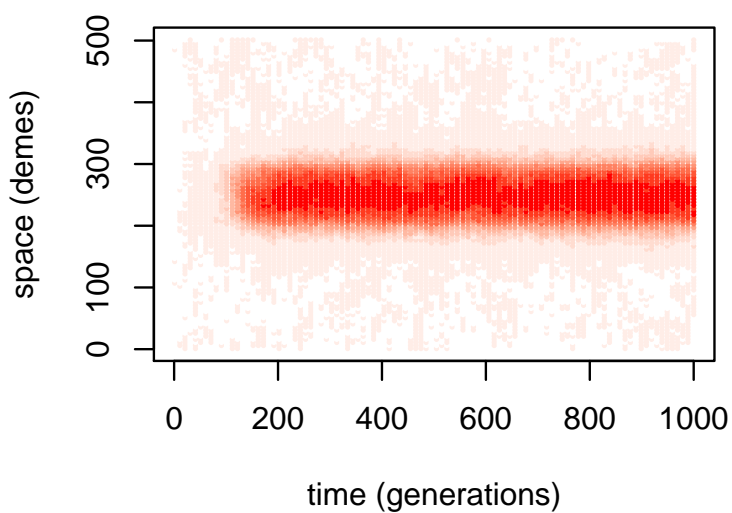

$s_m = 0.01, s_p = 0.00231$

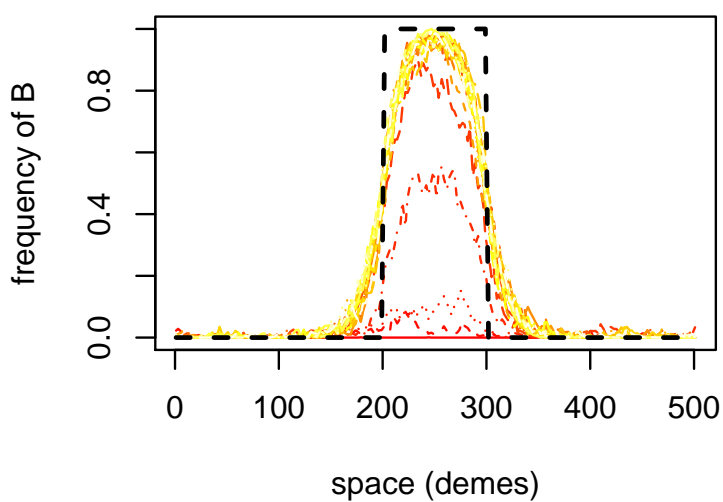

$\rho = 600, \mu = 1e-05$

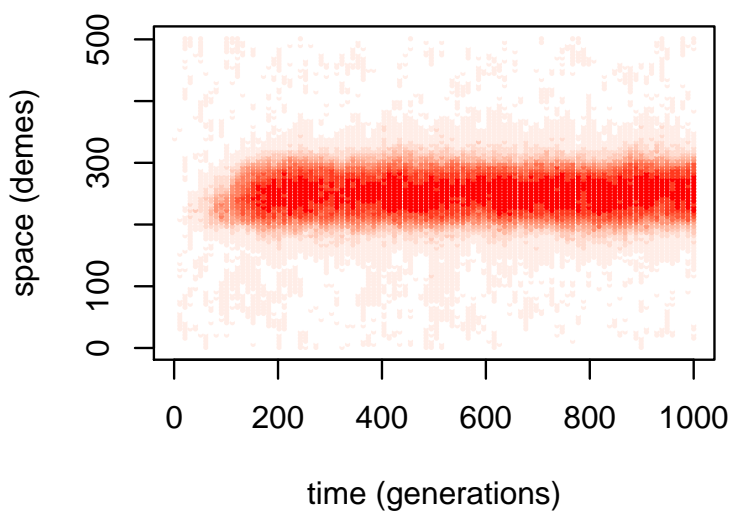

$s_m = 0.01, s_p = 0.00231$

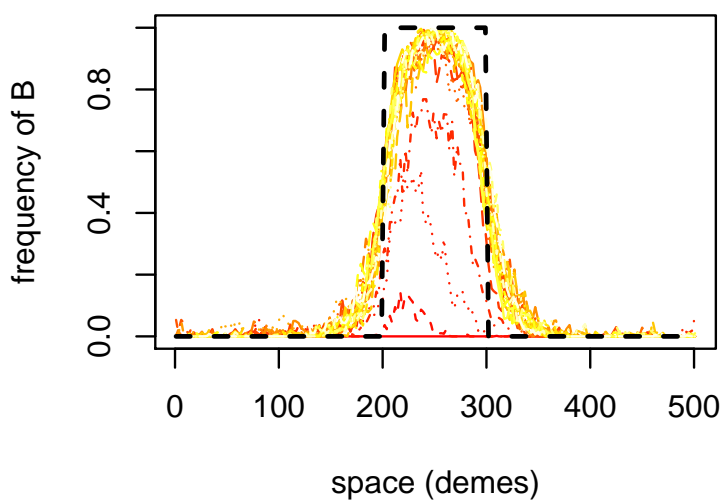

$\rho = 50, \mu = 1e-05$

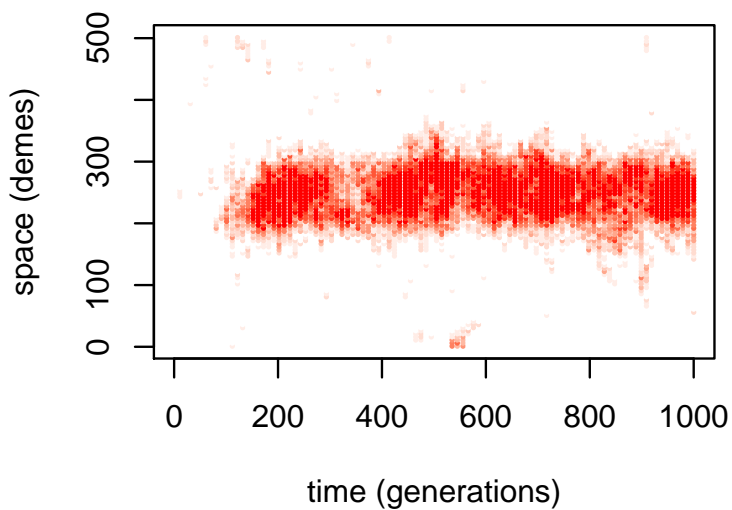

$s_m = 0.01, s_p = 0.00231$

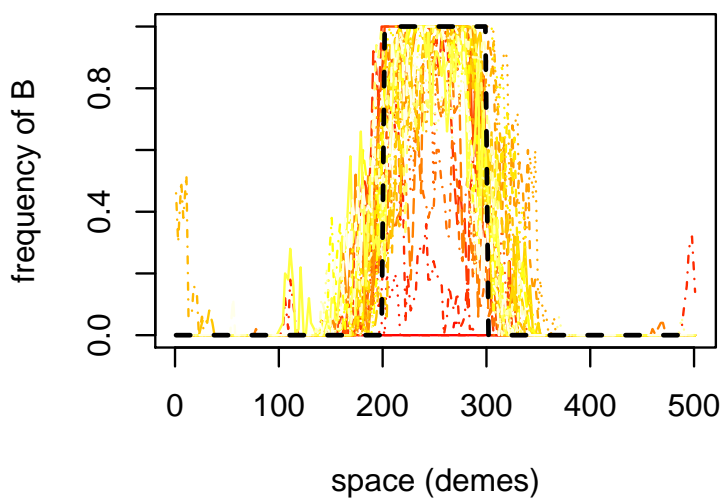

Supplement: S4 Fig — On the left of each is a space-time heatmap of the local frequency of B alleles; and on the right are twenty-five curves showing the frequencies of B at evenly spaced time points (i.e., each line represents a vertical slice through the plot on the left); dotted black lines indicate the patches where B is advantageous. (PDF) [file pgen.1005630.s008.pdf]

$\rho = 1200, \mu = 1e-05$

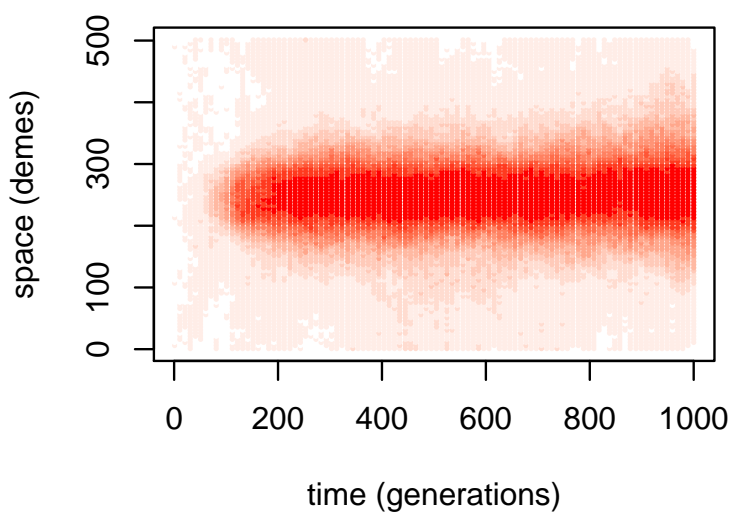

$s_m = 0.001, s_p = 0.00231$

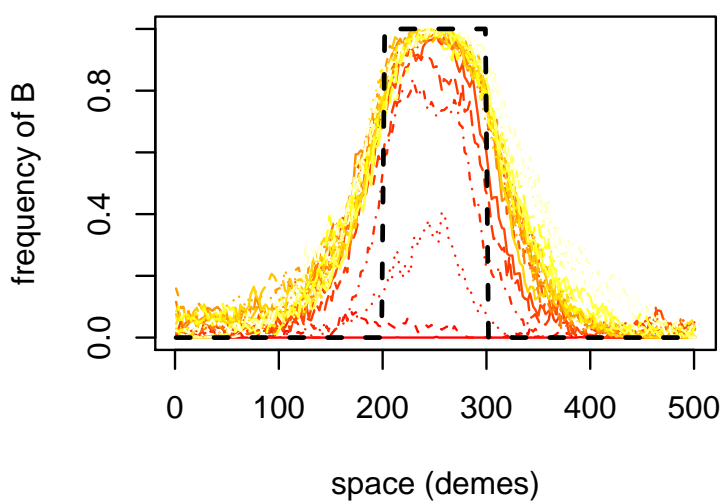

$\rho = 600, \mu = 1e-05$

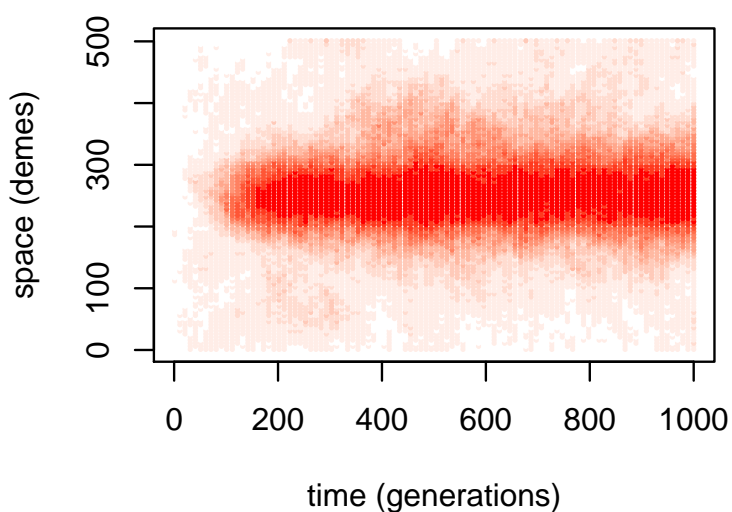

$s_m = 0.001, s_p = 0.00231$

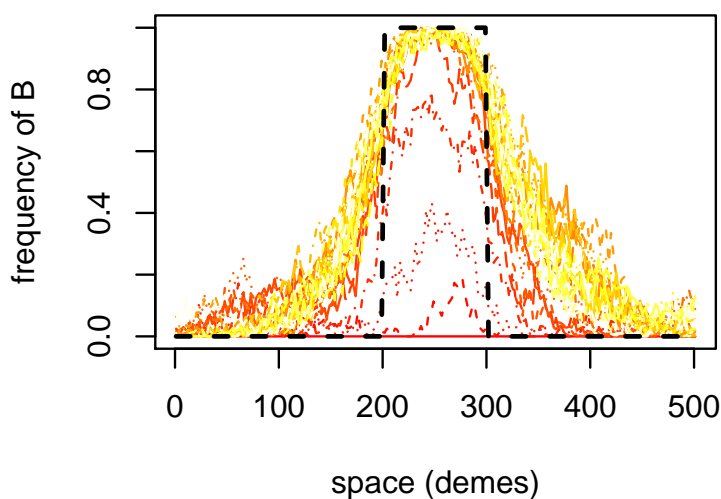

$\rho = 50, \mu = 1e-05$

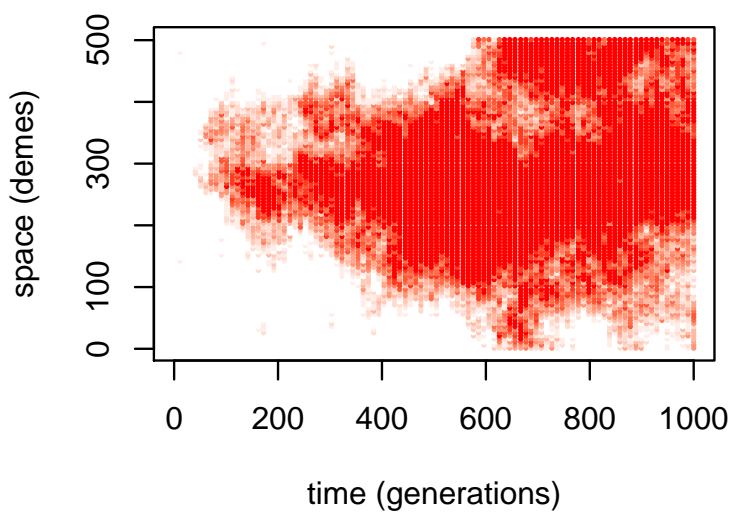

$s_m = 0.001, s_p = 0.00231$

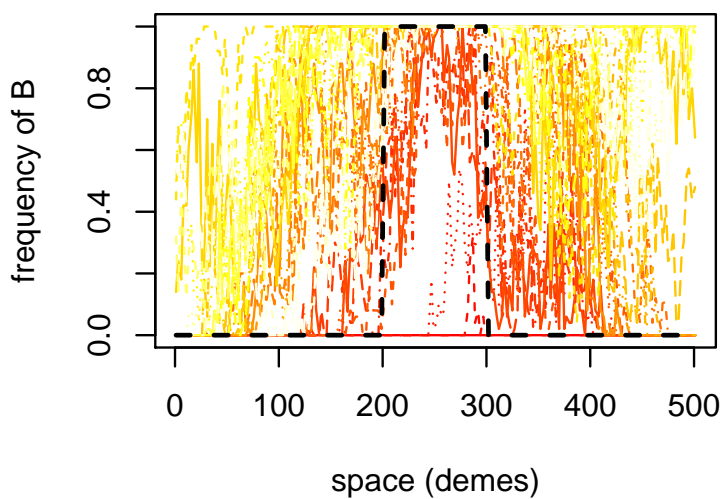

Supplement: S5 Fig — On the left of each is a space-time heatmap of the local frequency of B alleles; and on the right are twenty-five curves showing the frequencies of B at evenly spaced time points (i.e., each line represents a vertical slice through the plot on the left); dotted black lines indicate the patches where B is advantageous. (PDF) [file pgen.1005630.s009.pdf]

$\rho = 1600, \mu = 1e-05$

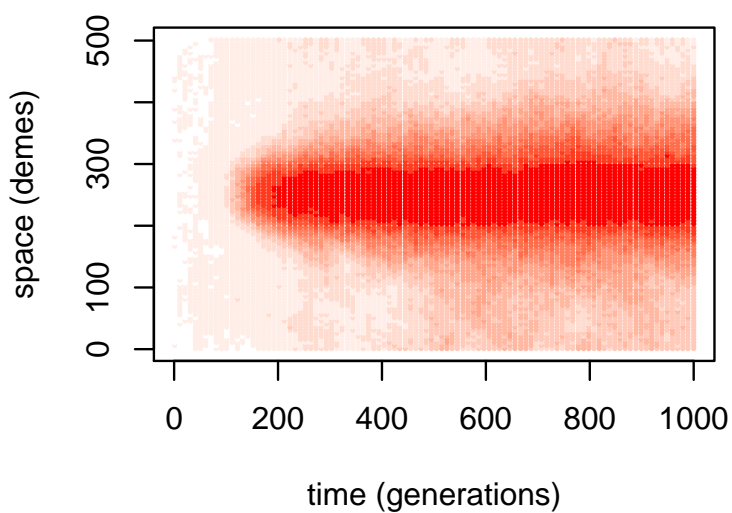

$s_m = 1e-04, s_p = 0.00231$

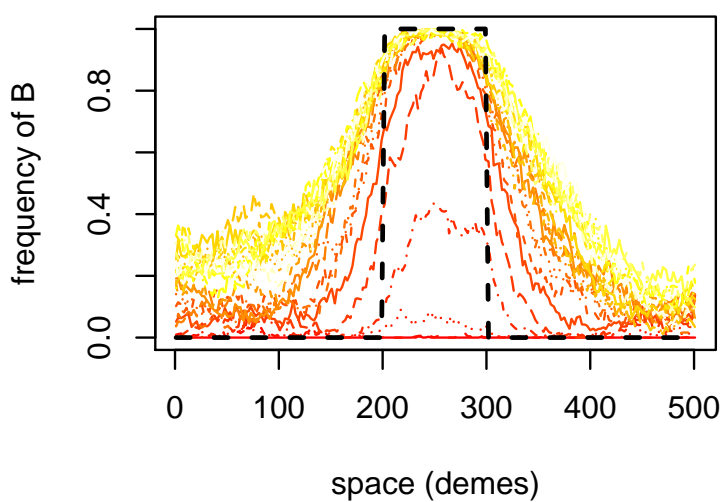

$\rho = 400, \mu = 1e-05$

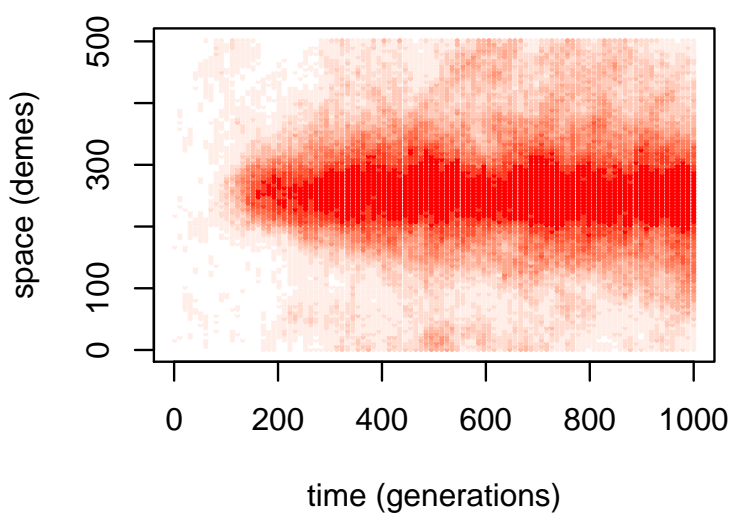

$s_m = 1e-04, s_p = 0.00231$

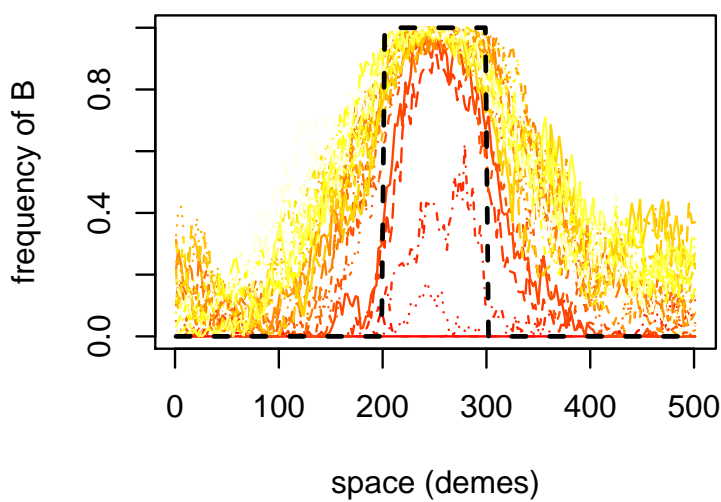

$\rho = 25, \mu = 1e-05$

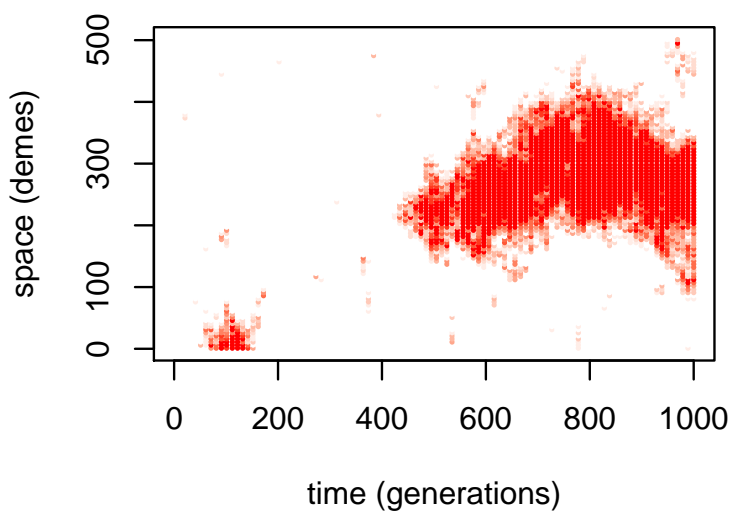

$s_m = 1e-04, s_p = 0.00231$

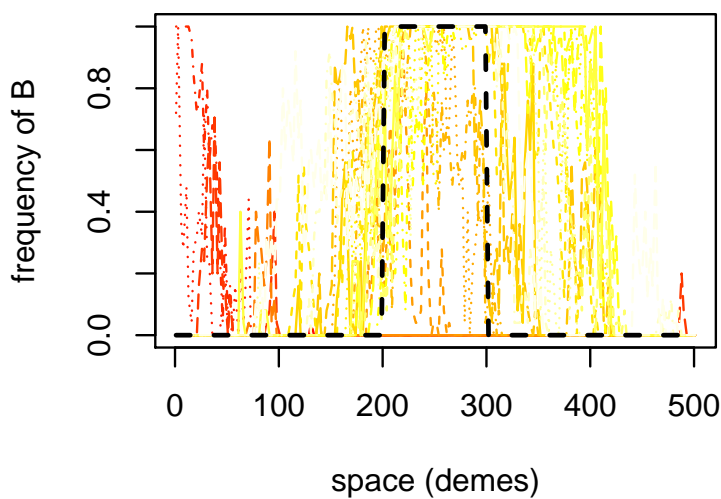

Supplement: S6 Fig — On the left of each is a space-time heatmap of the local frequency of B alleles; and on the right are twenty-five curves showing the frequencies of B at evenly spaced time points (i.e., each line represents a vertical slice through the plot on the left); dotted black lines indicate the patches where B is advantageous. (PDF) [file pgen.1005630.s010.pdf]

$\rho = 4000, R = 80$

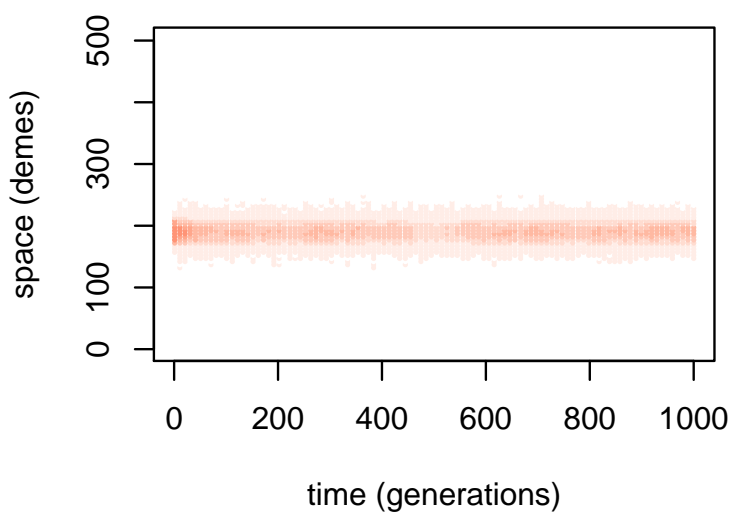

$s_m = 0.1, s_p = 0.00231$

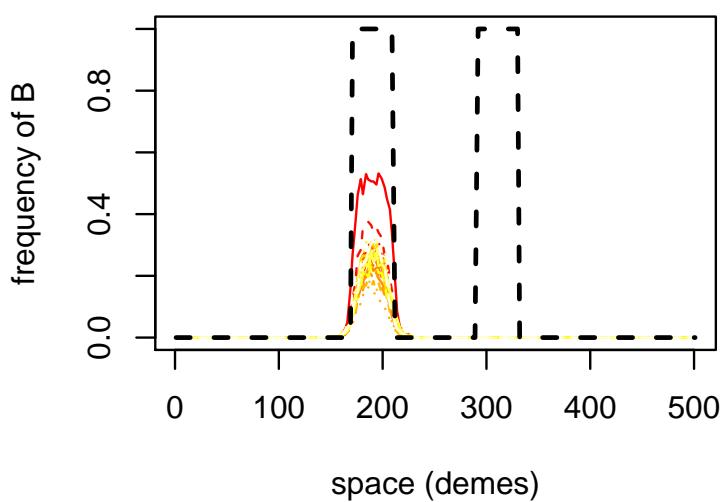

$\rho = 1000, R = 80$

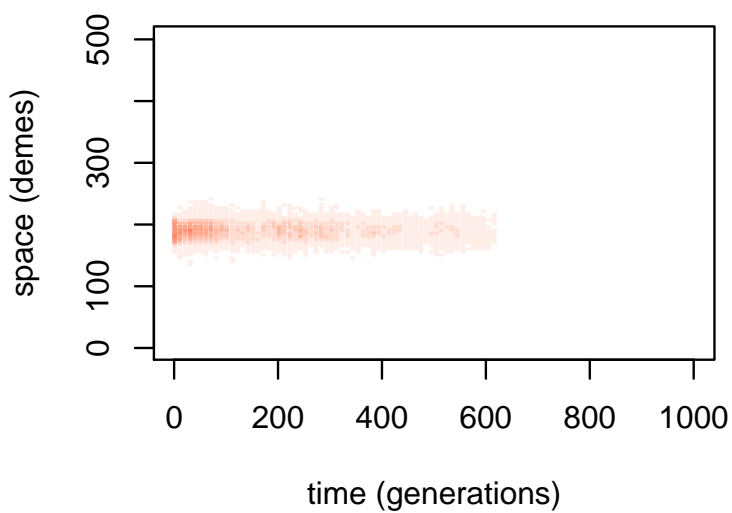

$s_m = 0.1, s_p = 0.00231$

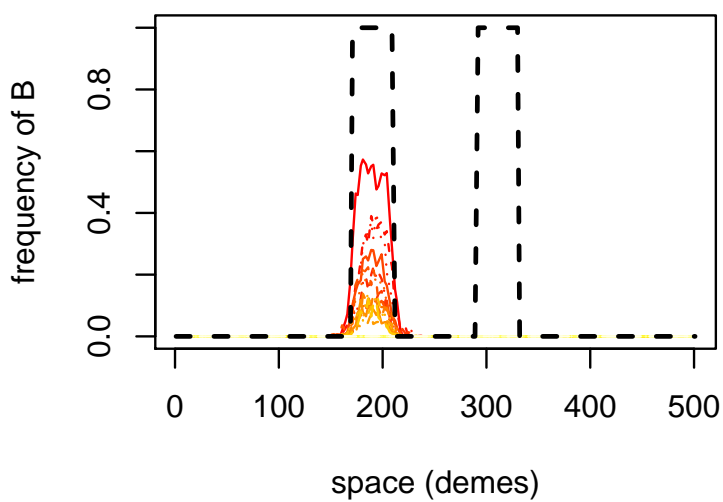

$\rho = 100, R = 80$

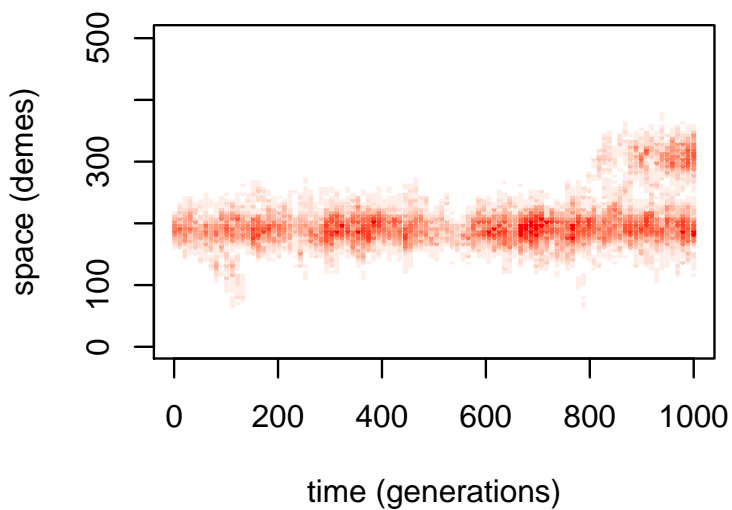

$s_m = 0.01, s_p = 0.00231$

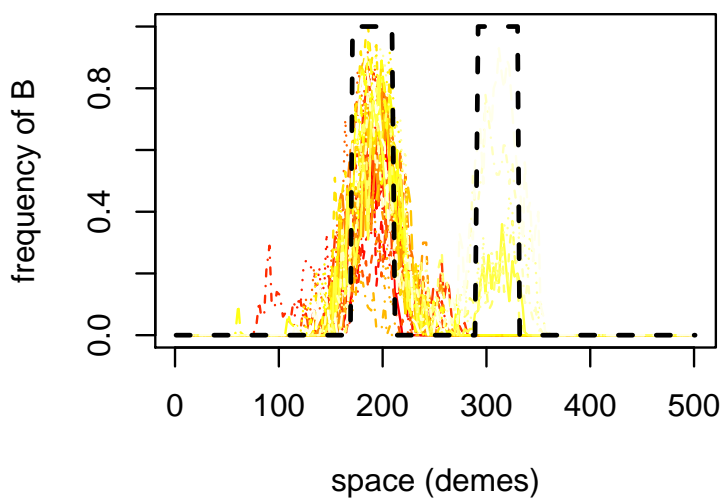

Supplement: S7 Fig — On the left of each is a space-time heatmap of the local frequency of B alleles; and on the right are twenty-five curves showing the frequencies of B at evenly spaced time points (i.e., each line represents a vertical slice through the plot on the left); dotted black lines indicate the patches where B is advantageous. (PDF) [file pgen.1005630.s011.pdf]

$\rho = 4000, R = 80$

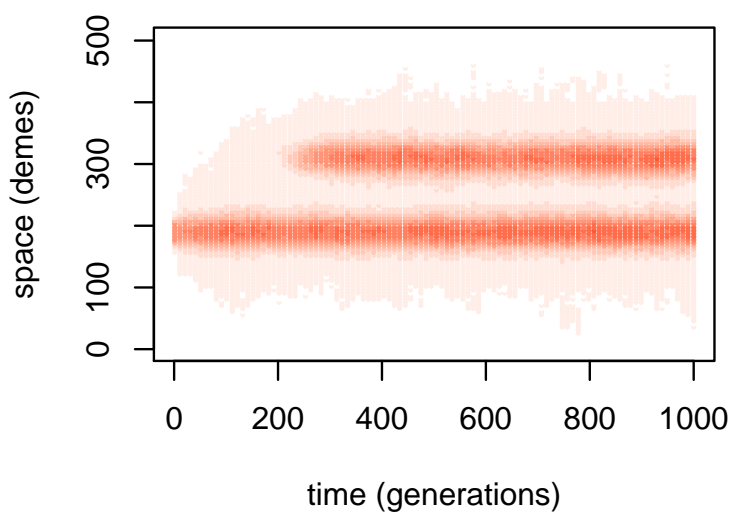

$s_m = 0.01, s_p = 0.00231$

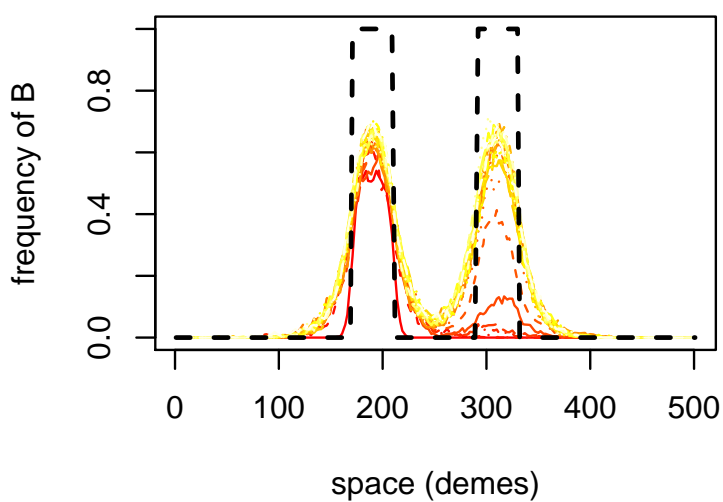

$\rho = 1000, R = 80$

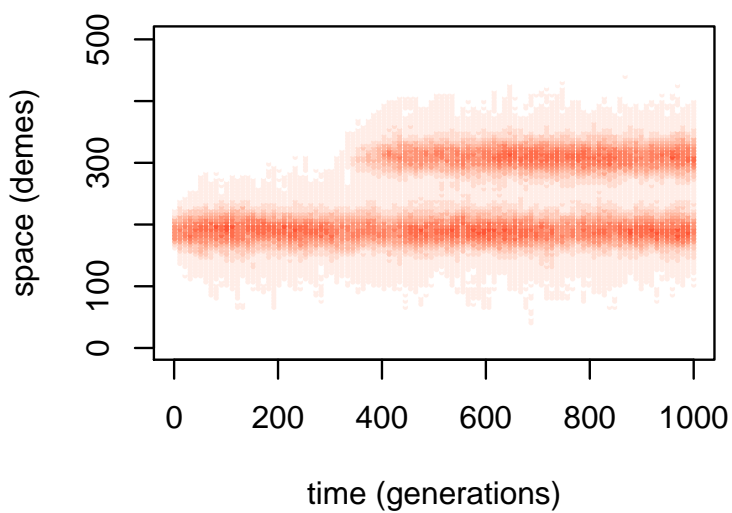

$s_m = 0.01, s_p = 0.00231$

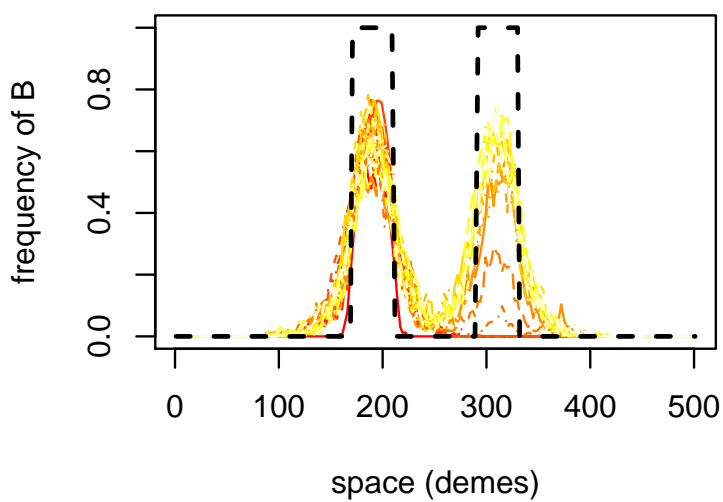

$\rho = 100, R = 80$

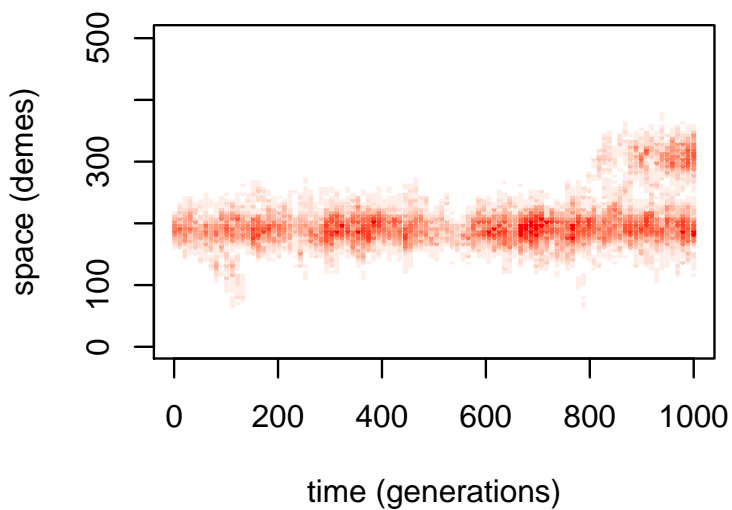

$s_m = 0.01, s_p = 0.00231$

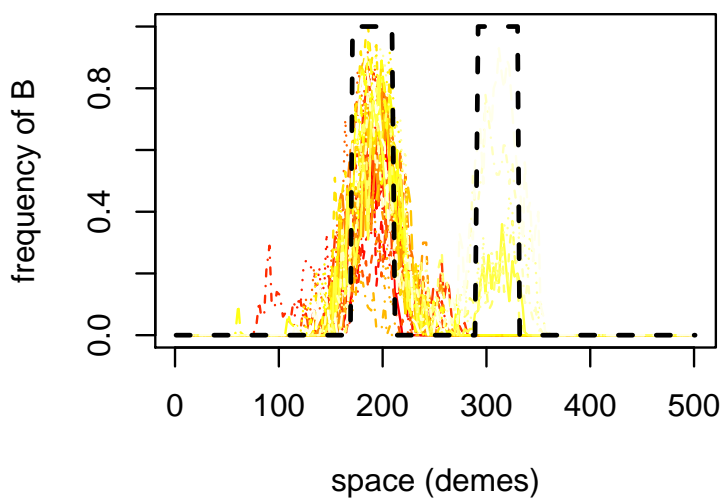

Supplement: S8 Fig — On the left of each is a space-time heatmap of the local frequency of B alleles; and on the right are twenty-five curves showing the frequencies of B at evenly spaced time points (i.e., each line represents a vertical slice through the plot on the left); dotted black lines indicate the patches where B is advantageous. (PDF) [file pgen.1005630.s012.pdf]

$\rho = 4000, R = 80$

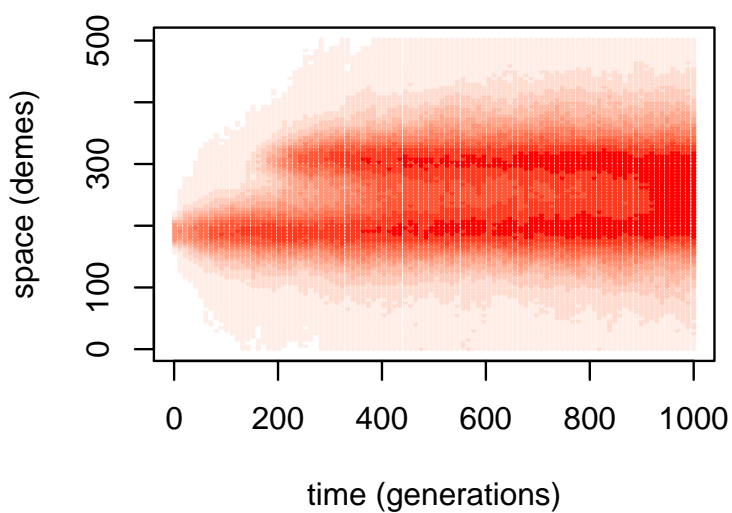

$s_m = 0.001, s_p = 0.00231$

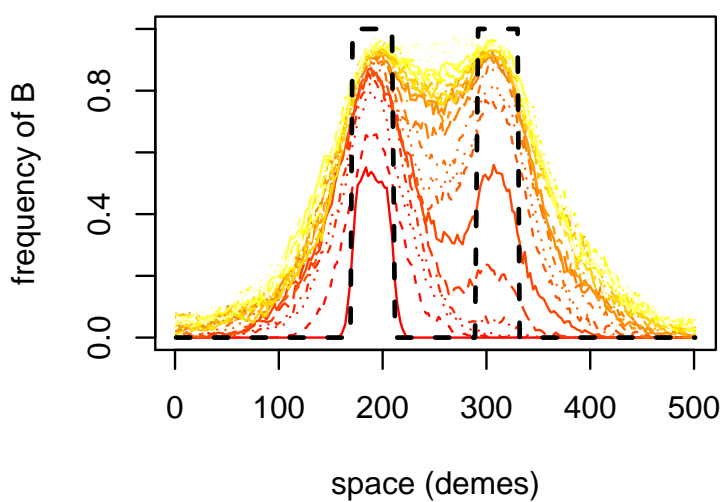

$\rho = 1000, R = 80$

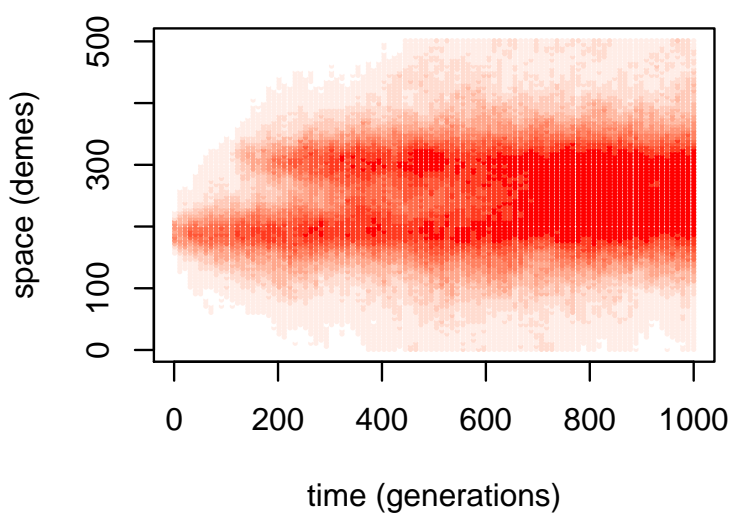

$s_m = 0.001, s_p = 0.00231$

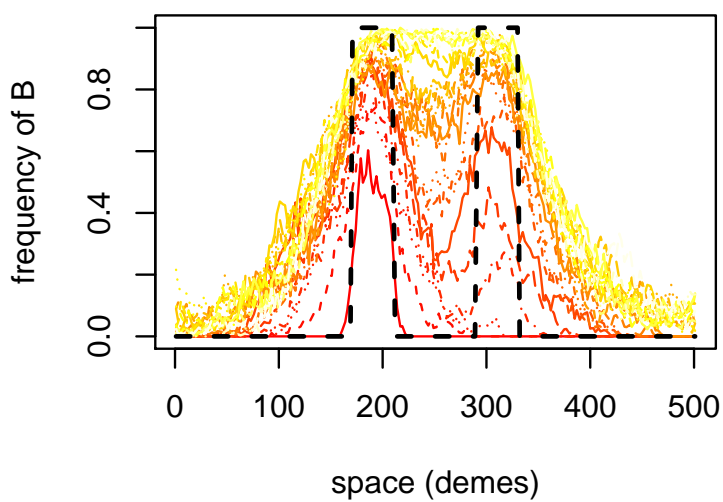

$\rho = 100, R = 80$

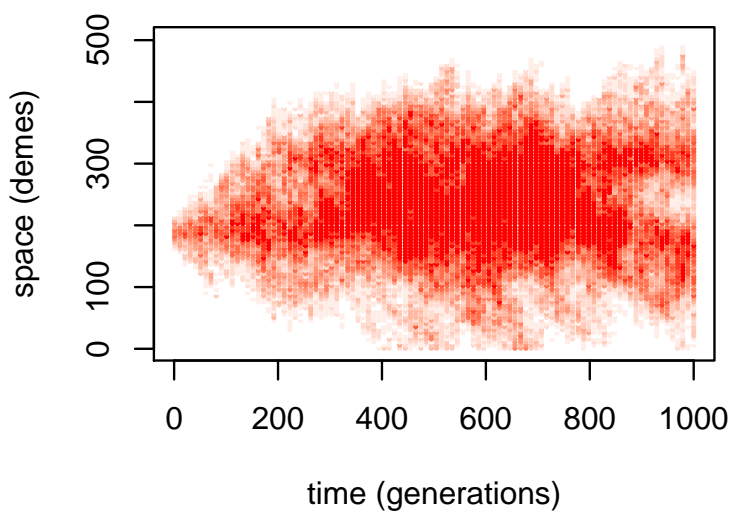

$s_m = 0.001, s_p = 0.00231$

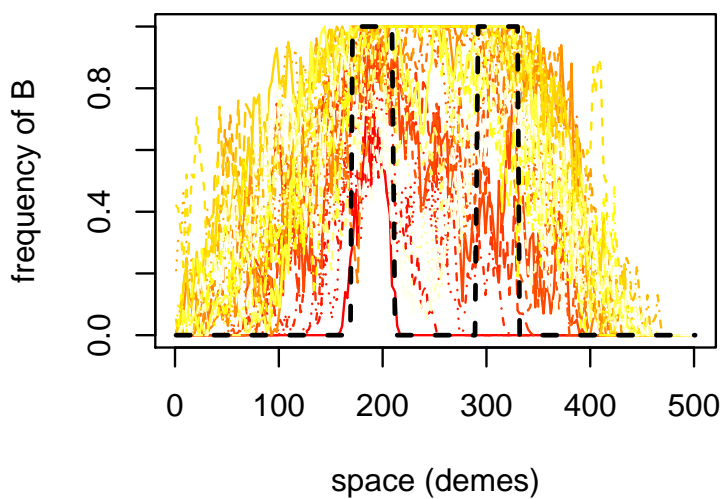

Supplement: S9 Fig — On the left of each is a space-time heatmap of the local frequency of B alleles; and on the right are twenty-five curves showing the frequencies of B at evenly spaced time points (i.e., each line represents a vertical slice through the plot on the left); dotted black lines indicate the patches where B is advantageous. (PDF) [file pgen.1005630.s013.pdf]

$\rho = 4000, R = 80$

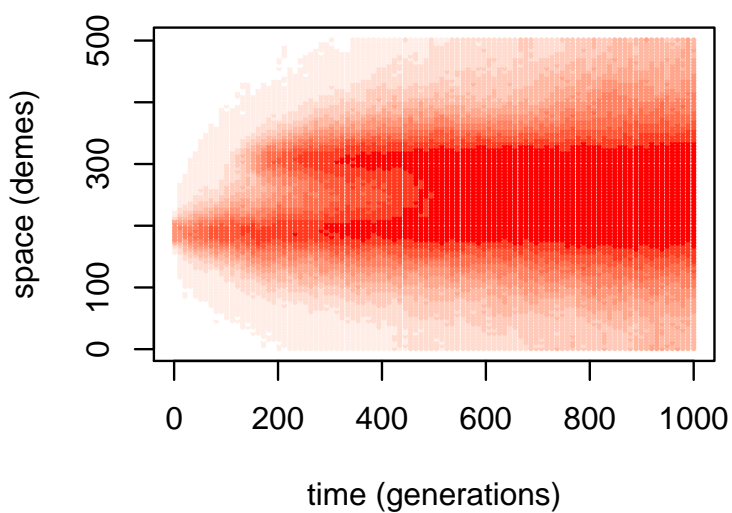

$s_m = 1e-04, s_p = 0.00231$

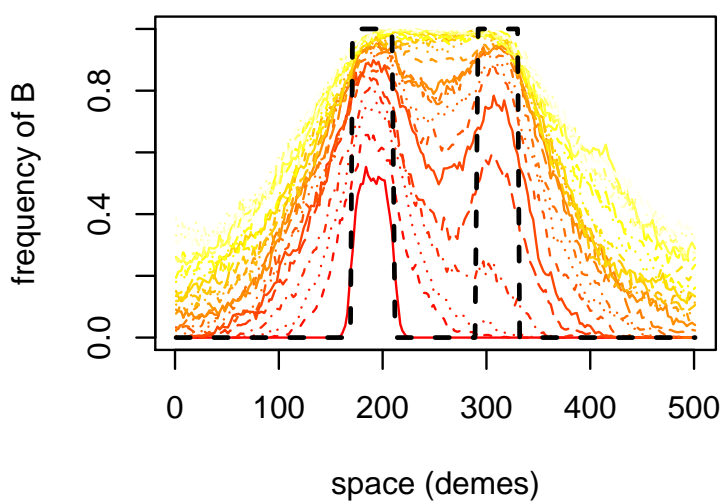

$\rho = 1000, R = 80$

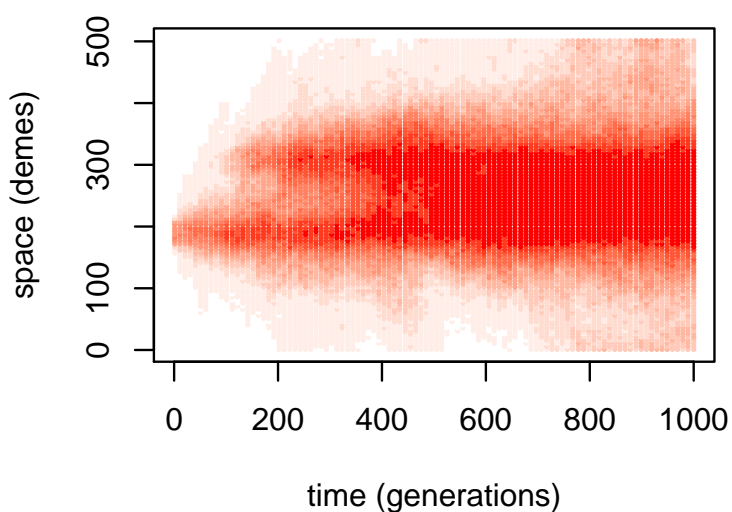

$s_m = 1e-04, s_p = 0.00231$

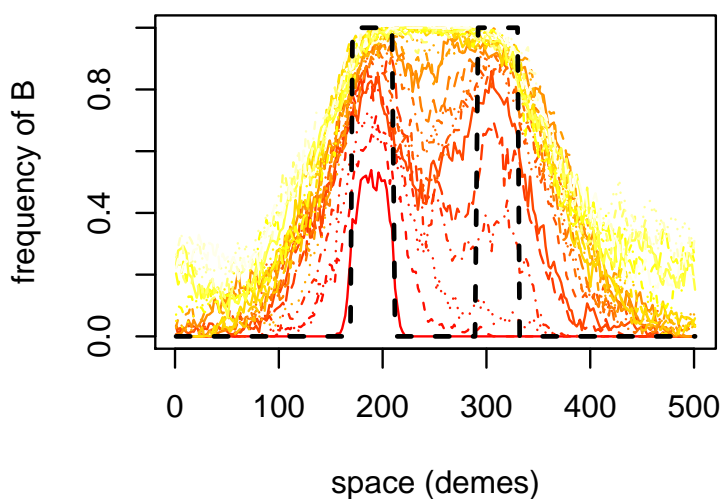

$\rho = 100, R = 80$

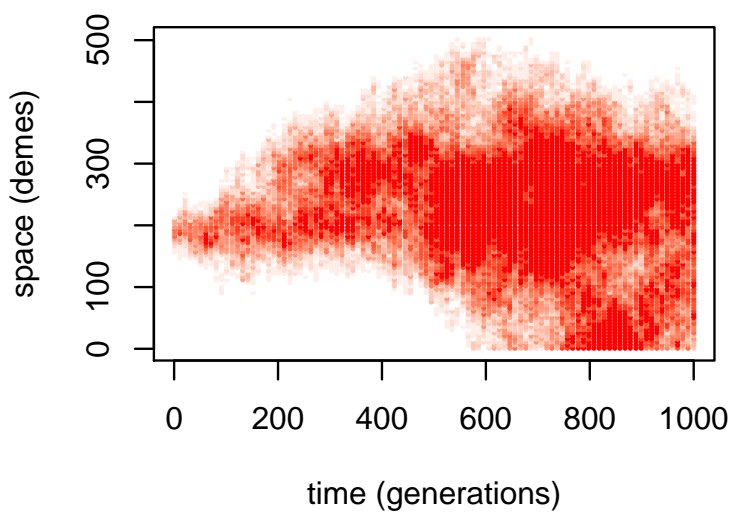

$s_m = 1e-04, s_p = 0.00231$

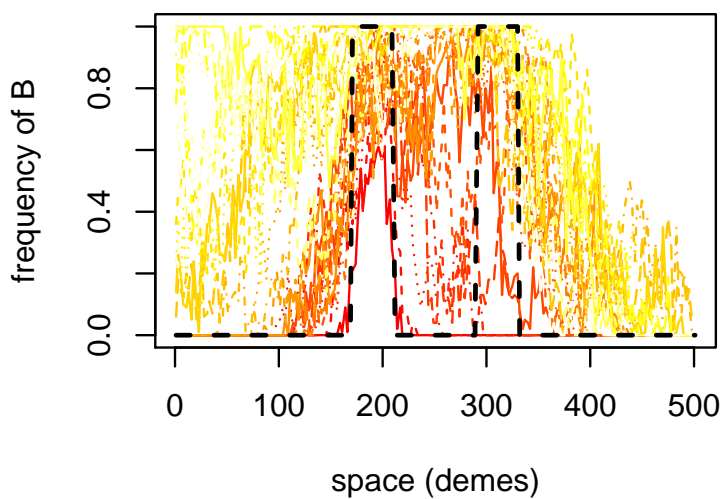

Supplement: S10 Fig — On the left of each is a space-time heatmap of the local frequency of B alleles; and on the right are twenty-five curves showing the frequencies of B at evenly spaced time points (i.e., each line represents a vertical slice through the plot on the left); dotted black lines indicate the patches where B is advantageous. (PDF) [file pgen.1005630.s014.pdf]
